# Multimodal Collaborative Modeling of Molecular Structures and Biomedical Text for Accurate Drug–Drug Interaction Extraction †

**DOI:** 10.3390/biomedicines14061231

**Published:** 2026-05-29

**Authors:** Liumei Yang, Yiyang Shi, Fangfang Han, Yongming Cai

**Affiliations:** 1 School of Medical Information and Engineering, Guangdong Pharmaceutical University, Guangzhou 510006, China; 2112359011@stu.gdpu.edu.cn (L.Y.); syy528000@126.com (Y.S.); 2 NMPA Key Laboratory for Technology Research and Evaluation of Pharmacovigilance, Guangzhou 510006, China; 3 Guangdong Provincial Traditional Chinese Medicine Precision Medicine Big Data Engineering Technology Research Center, Guangzhou 510006, China

**Keywords:** drug–drug interactions (DDIs), multimodal learning, biomedical text mining

## Abstract

**Background**: Drug–drug interactions (DDIs) account for about 30% of adverse drug reactions and 5–10% of hospital deaths. Combination therapy increases DDI risks, yet extracting DDIs from biomedical text remains challenging: existing methods rely on surface co-occurrence and fail when multiple drugs and interactions coexist in a sentence. Prior multimodal approaches simply concatenate text, molecular, or knowledge features without deep alignment, leading to misclassification of structurally similar but non-interacting drug pairs. **Methods**: We propose MultiMod-DDI, a framework that constructs a ternary evidence chain of “molecular structure–biological entities–DDI text”. Unlike existing work, MultiMod-DDI introduces (1) PS-AEGNN, a molecular graph network with ProbSparse self-attention to capture long-range chemical dependencies; (2) an adaptive position interaction vector that dynamically weights distant semantic links between drug entities; and (3) a multi-stage adaptive fusion module that sequentially applies subgraph-molecule attention and text-guided gating. These components are co-designed to enforce structured semantic alignment among heterogeneous modalities, effectively addressing the specific challenge of matching drug pairs to their correct interaction types in complex, multi-drug sentences. **Results**: On SemEval-2013 Task 9, MultiMod-DDI achieves 85.57% *F1_macro_* and 85.20% *F1_micro_*, outperforming state-of-the-art models. **Conclusions**: Through multimodal deep semantic alignment, MultiMod-DDI effectively resolves the mismatch between drug pairs and their interaction types in complex biomedical texts. The integration of multimodal features greatly improves DDI extraction accuracy, offering a reliable method for intelligent DDI mining from biomedical literature.

## 1. Introduction

In the treatment of complex disorders, drug combination therapy has shown notable benefits [1]; however, the drug–drug interactions (DDIs) triggered by multidrug regimens have become a critical clinical safety concern, as such interactions may alter the toxicity or efficacy of one or more drugs [2,3]. Therefore, comprehensively extracting information related to DDIs from the scientific literature is particularly critical for preventing risks associated with polypharmacy [4]. Automated DDI information extraction not only reduces the workload of medical professionals in literature reviews but also enables efficient identification of potential drug interactions, making accurate classification of DDI types essential for ensuring medication safety [5,6]. With the rapid growth of the biomedical literature, traditional manual annotation and database maintenance approaches can no longer meet the requirements for effective DDI mining from massive textual data [7]. Consequently, developing more advanced automated tools and methods for extracting valid DDI information is of great importance to improve the safety and efficacy of clinical treatment, better protect patients from adverse drug interactions, and further promote progress in drug development and clinical therapy [8].

Early statistical and rule-based methods (e.g., CRF, SVM) relied on manual features and struggled with the complexity of biological texts [9]. For instance, Bhasuran et al. [10] improved gene-disease extraction via ensemble learning, but generalization remained limited. While CNNs, RNNs, LSTMs, and BiLSTMs facilitated local feature extraction [11,12] and Transformers captured global semantics [13], early deep learning models still struggled with complex semantic understanding. To overcome this, the field shifted to pre-training and fine-tuning. BERT [14] learned rich linguistic representations from large corpora. Researchers then incorporated drug molecular structures and knowledge graphs into pre-training. Asada et al. [15] added molecular structures but missed structured entity relationships. Duan et al. [16] introduced attention and molecular features yet lacked deep cross-modal alignment, causing misclassification of structurally similar but non-interacting drug pairs. Shi et al. [17] used SubAGCN to extract knowledge subgraphs with PubMedBERT [18] but omitted molecular structure, limiting chemical interpretability.

In parallel with extraction-oriented research, the DDI prediction field has advanced rapidly via graph-based deep learning. Methods such as HAN-DDI [19] and MPHGCL-DDI [20] leverage heterogeneous graph attention and contrastive learning to model drug relationships from molecular and knowledge data. However, these approaches target DDI prediction (inferring potential interactions from multimodal profiles) rather than DDI extraction (classifying explicitly stated interactions from biomedical text). Unlike prediction, extraction requires handling complex sentences, multiple drug mentions, and context-dependent semantic reasoning [21]. Our work focuses exclusively on DDI extraction, adapting multimodal ideas to fit text-centric challenges.

Most existing extraction methods capture only surface-level cues and fail to model deep entity logic in multi-drug sentences. They typically treat molecular structure, biomedical entities, and text as independent features rather than interlocking evidence layers. A drug’s molecular structure defines its chemical potential, biological entities mediate pharmacokinetic effects, and text describes clinical outcomes. Effective extraction thus requires synergistic multimodal modeling rather than simple concatenation. While our framework does not perform explicit causal reasoning, it aligns chemical and biological knowledge under textual guidance to distinguish true interactions from spurious co-occurrences.

Therefore, to address this cross-modal alignment challenge, we propose MultiMod-DDI. Unlike mere system integration, the true methodological novelty lies in three task-specific adaptations: (1) PS-AEGNN repurposes ProbSparse self-attention from time-series forecasting to molecular graphs, enabling sparse long-range dependency capture without quadratic cost; (2) the adaptive position interaction vector introduces a dynamically calibrated intensity range (0.9–1.1) specifically to suppress bystander-drug noise; (3) the multi-stage fusion module enforces a sequential order (subgraph-molecule attention followed by text-guided gating), grounded in the information bottleneck principle. These designs are purpose-built for multi-drug, multi-interaction sentences, not interchangeable with off-the-shelf components. We demonstrate that this principle-driven integration substantially outperforms state-of-the-art models.

Here, an “evidence chain” is defined as a structured, layer-by-layer aggregation of multimodal knowledge, where each modality provides a distinct but interdependent type of evidence that collectively supports the classification of a drug–drug interaction. The chain is “ternary” because it explicitly links three layers in a fixed order: chemical properties → biological pathways/targets → clinical or mechanistic descriptions in text.

A preliminary version of this work was presented at the 2nd International Conference on Biomedical Engineering and Medical Devices (ICBEMD 2026) and published in its proceedings as “MultiMod-DDI: A drug–drug interaction prediction model based on the ternary evidence chain of ‘molecular structure–biological entities–DDI text’” [22]. This manuscript significantly extends our conference paper in multiple dimensions. First, new experiments are introduced: (1) comprehensive comparisons with additional state-of-the-art models; (2) an ablation study on the intensity range of the adaptive position interaction vector; (3) a systematic evaluation of different feature fusion strategies; and (4) a quantitative analysis of inference efficiency (GPU memory, training time, parameter overhead). Second, new analytical insights are provided: a stratified evaluation on sentences with varying numbers of drug mentions, and a case study demonstrating how multimodal evidence corrects text-only misclassifications. Third, the methodological description of PS-AEGNN is substantially deepened, including full derivations of the ProbSparse self-attention formulation and its integration into molecular graph learning. Fourth, we introduce a multi-focal loss formulation to address class imbalance, which was not present in the conference version. Collectively, these extensions transform the conference proof-of-concept into a comprehensive journal-ready study, providing both quantitative and qualitative evidence of the framework’s robustness, generalizability, and practical efficiency.

Experiments on the SemEval-2013 Task 9 dataset show that MultiMod-DDI achieves ${F1}_{macro}$ and ${F1}_{micro}$ of 85.57% and 85.20%, respectively, significantly outperforming current state-of-the-art models and confirming the effectiveness of multimodal collaborative modeling in improving DDI extraction accuracy.

## 2. Materials and Methods

### 2.1. Dataset

Training data were obtained from the SemEval-2013 Task 9 dataset [6]. This dataset has been widely used to develop and evaluate automated methods for biomedical text-based drug named entity recognition and drug–drug interaction (DDI) extraction. Data were collected from two public resources: DrugBank and MedLine. In total, 730 DDI-related articles were retrieved from DrugBank and 175 from MedLine. All target drugs and their corresponding interaction types were manually annotated by domain experts. The dataset was then split into training and test sets following the official partitioning scheme. SemEval-2013 Task 9 [6] defines five DDI categories for classification: “Advice”, “Effect”, “Mechanism”, “Interaction”, and “Other”. Detailed dataset statistics under the official train/test split are presented in Table 1.

The following is the DDI-type labelling specification for this dataset:

- Mechanism: Describes the pharmacokinetic mechanism of drug interactions. Ep: Median gastric pH was significantly higher when indinavir was taken after didanosine administration;
- Effect: Enhancement or diminution of the effect of a drug on another drug. Ep: Verapamil also significantly decreased the incidence of lymphatic invasion in adenocarcinomas, an effect that was enhanced by bombesin.
- Advice: Clinical Management Recommendations for Drug Interactions. Ep: Barbiturates and glutethimide should not be administered to patients receiving coumarin drugs.
- Interaction: Indicates the presence of interactions but provides no specific description. Ep: The drug interaction between warfarin and rifampin is not well-known.
- Other: Indicates only that there may be no significant interactions not specifically described. Ep: Cysteine was covalently linked to carbodiimide activated nacmc.

The dataset exhibits severe class imbalance, with a large majority of negative samples and a scarcity of positive samples. To alleviate this issue, we employ a hybrid strategy combining undersampling and oversampling on the training set. Specifically, undersampling is used to remove redundant duplicate negative samples, while oversampling increases the number of rare positive samples to strengthen their contribution during model training.

### 2.2. Data Preprocessing

Each drug pair in a sentence is a potential relation instance. Previous research has demonstrated that drug names have a modest contribution to DDI classification, with key semantics relying on contextual interactions rather than individual drug names [11]. Therefore, in this study, the target drug pairs in each case were labeled as “DRUG1” and “DRUG2”, respectively, while the remaining medicines were universally substituted with “DRUGOTHER” to remove irrelevant naming interference and focus on essential semantic structures. Taking the preprocessing example in Table 2 as an example, the original sentence “Antacids increase the rate of absorption of pseudoephedrine, while kaolin decreases it.” is transformed to “DRUG1 increases the rate of absorption of DRUG2, while DRUGOTHER decreases it.” The process not only preserves the entity location information but also improves the model’s generalization to cross-drug pair patterns by standardizing the naming ability.

### 2.3. Overview of MultiMod-DDI

**Figure 1:**
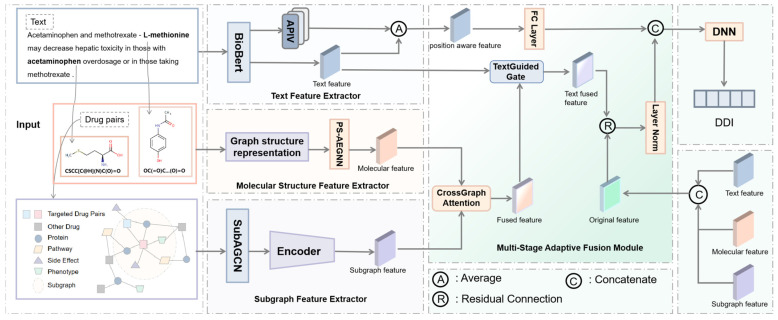
This figure illustrates MultiMod-DDI workflow, where APIV represents the adaptive position interaction vector mechanism.

The overall architecture of MultiMod-DDI is shown in Figure 1. BioBERT [13] encodes textual semantics and drug position embeddings, enhanced by an adaptive position interaction vector to capture long-range entity dependencies. For molecular structures, we propose PS-AEGNN, a graph neural network with ProbSparse self-attention [23] to model long-range chemical interactions. SubAGCN [17] extracts task-relevant subgraphs from a biomedical knowledge graph. A multi-stage adaptive fusion module then integrates these three modalities via subgraph and molecule cross-attention and text-guided gating, producing a joint representation for DDI classification.

Finally, to achieve effective DDI extraction from text, knowledge subgraphs, and molecular characteristics, we design a multi-stage adaptive fusion module. This module introduces a cross-modal attention mechanism as well as a dynamic gating mechanism to achieve deep semantic alignment between distinct modalities. This approach enables the model to dynamically alter the fusion ratio of molecular structure and knowledge subgraph features in response to text features, ensuring that information from each modality is adequately represented in the final joint representation. In this way, our framework establishes a multilevel evidence chain comprising “molecular structure–biological entities–DDI text,” providing more comprehensive support for the accurate classification of DDI types. The details of the model are elaborated in the subsequent sections.

#### 2.3.1. Drug–Drug Interaction Text Feature Extraction

The details of the model are elaborated in the subsequent sections. In this model, BioBERT [13] is used to extract textual aspects linked to DDI. BioBERT, a biomedical domain-specific pre-trained language model, is built on the BERT architecture and pre-trained using domain-specific data, such as PubMed abstracts and PMC full text, to solve the issue of insufficient semantic comprehension in general-purpose models for biomedical texts.

DDI relationship extraction involves specialized terminology and complicated phrases, and typical NLP models struggle to effectively capture drug names, chemical components, and semantic interactions. In contrast, BioBERT can better capture drug names, chemical substances, and their semantic relationships through pre-training on a biomedical corpus. Given a sequence $\left(S=\{x_{1},x_{2},\ldots ,x_{n}\}\right)$ as input, where $x_{i}$ is the i-th token and n is the length of the sequence, each layer l (1≤l≤L) of BioBERT outputs a hidden state matrix $\;H^{l}$:

(1) $$H^{l}=TransformerLayer\left(H^{\left(l-1\right)}\right)$$

where $H^{l}\in R^{n\times d}$, and d is the dimension of the hidden state. Specific features are extracted from the last layer of hidden states. The hidden states labeled with [CLS] are usually used as a representation of the whole sentence:

(2) $$h_{\;[CLS]\;}=H^{L}[0]$$

where $H^{L}$ is the hidden state of the last layer, and L is the total number of layers. So, the final text description representation $h_{text}$ is obtained as

(3) $$h_{text}=h_{\;[CLS]\;}$$

Notably, the token embeddings and location embeddings output by BioBERT not only provide the basis for textual semantic modeling but also lay the foundation for the subsequent generation of adaptive position interaction vectors. Specifically, the word embedding $e_{i}^{token}$ of each token encodes the lexical semantics, while the positional embedding $e_{i}^{pos}$ captures the character-level positional information, which together constitute the underlying feature representation of the text.

#### 2.3.2. Adaptive Position Interaction Vector

To address the challenge of matching drug pairs to their correct interaction types in multi-drug sentences, we design an adaptive position interaction vector (APIV) upon BioBERT’s embeddings. APIV dynamically adjusts intensity to quantify positional relations and semantic associations between tokens and drug entities, automatically prioritizing target drug pairs while suppressing interference from irrelevant drugs and redundant context. The design of the adaptive position interaction vector (APIV) is theoretically grounded in relative position encoding and distance-decayed context aggregation, two widely validated principles for relational modeling in textual data. Instead of relying on ad hoc empirical tuning, APIV implements a lightweight and interpretable contextual attention prior that enhances semantic contributions near drug entities while gradually decaying weights for distant tokens. This design follows a well-supported inductive bias in DDI extraction: relational evidence is spatially concentrated around drug mentions, and contextual relevance diminishes with increasing distance.

Analysis of extensive biomedical text data indicates that key DDI evidence is often implicitly encoded in the contextual span between two drug entities. For instance, Duan et al. [16] statistically analyzed entity-level interaction patterns in the SemEval-2013 Task 9 dataset, as summarized in Table 3. The results confirm that a large proportion of samples conform to this implicit contextual pattern. Accordingly, our proposed method better captures the semantic correspondence between drug pairs and their associated interaction types, effectively improving the accuracy and reliability of DDI extraction.

First, we locate the starting and ending indexes of the drug entities DRUG1 and DRUG2 after converting the sentences into token sequences using a tokenizer:

(4) $$\begin{array}{l} entity\_index\\ =\left[i_{\;DRUG1\_start},i_{\;DRUG1\_end},i_{\;DRUG2\_start},i_{\;DRUG2\_end}\right]\\ i_{\;DRUG1\_end}=i_{\;DRUG1\_start}+L\left(DRUG1\right)\\ i_{\;DRUG2\_end}=i_{\;DRUG2\_start}+L\left(DRUG2\right) \end{array}$$

where $L\left(DRUG1\right)$ and $L\left(DRUG2\right)$ are the lengths of the entities after word splitting. In DDI extraction, relational semantics is highly concentrated near drug entities, while tokens far from entities contribute weakly to relation judgment. A narrow range near 1.0 ensures that the model strengthens entity-centered semantics without causing weight distortion or instability. This design follows the principles of relative position encoding [24] and distance-decayed context aggregation [25], while the optimal intensity interval [0.9, 1.1] is determined empirically via Table 5. Since the entity itself is the core carrier of the interaction relationship, a higher range of weight fluctuation is given to its neighboring words to highlight the semantic focus. So, higher weight values are given to words that are in the range of the drug entity, where the equation calculates the weight value:

(5) $${intensity}_{i}=Uniform\left(0.9-t\cdot dis,1.1+t\cdot dis\right)$$

where *dis* denotes the ratio of the total length of the entity to the number of tokens of the sentence *N*, reflecting the space occupied by the entity in the sentence:

(6) $$dis=\frac{\left(i_{\;DRUG1\_end}-i_{\;DRUG1\_start}\right)+\left(i_{\;DRUG2\_end}-i_{\;DRUG2\_start}\right)}{N}$$

where *t* = 0.1 is the intensity adjustment factor, which controls the range of fluctuation in intensity values. Words in the non-entity range are adjusted for intensity values by calculating the distance to the nearest entity using the equation:

(7) $${intensity}_{i}=Uniform\left(0.3-t\cdot \frac{{context\_dis}_{i}}{N},0.5+t\cdot \frac{{context\_dis}_{i}}{N}\right)$$

This study uses relative position embedding on location information:

(8) $$\begin{array}{ll} {pos_{embedding}}_{i}= & [one-hot\left(i\epsilon DRUG1\_range\right),one\\ & -\;hot\left(i\epsilon DRUG2\_range\right),\frac{{context\_dis}_{i}}{N},{center\_dis}_{i}] \end{array}$$

**Figure 2:**
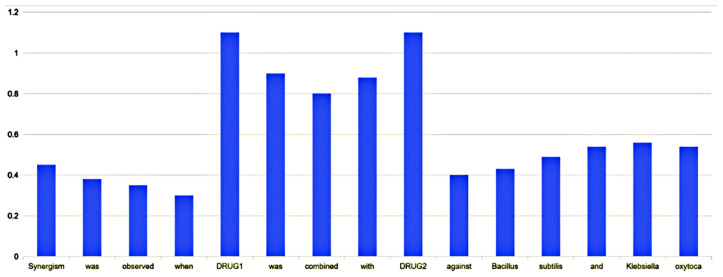
Visual representation of adaptive position interaction vectors.

Among them, the one-hot function is used to determine whether a word falls within the range of an entity or not, and the last two are normalized distance features. Dynamic intensity is interactively fused with position ${intensity}_{i}$, which serves as the attention weight. The word embedding $e_{i}^{token}$ is fused with position embedding $e_{i}^{pos}$. Figure 2 shows the output of this step, which generates an adaptive position interaction vector $v_{i}$ for the *i*-th word:

(9) $$v_{i}={intensity}_{i}\cdot \left(e_{i}^{token}+e_{i}^{pos}\right)$$

thus, for the set of all words ${\;v}_{i}$ in the sentence:

(10) $${V=[v}_{1},v_{2},\ldots ,v_{N}]=IntensityMatrix⊙\left(E^{token}+E^{pos}\right)$$

where IntensityMatrix is a diagonal matrix with diagonal elements of ${intensity}_{i}$.

The intensity range (0.9–1.1) is selected based on theoretical principles of stable attention normalization and empirical validation as demonstrated in Table 8. Values near 1.0 avoid excessive weighting fluctuation and gradient instability, while narrow bounds preserve entity-centered contextual focus without distorting semantic distributions. This choice is not heuristic but corresponds to the valid range of soft attention scaling in biomedical relation extraction.

Built on relative position encoding and distance-decayed context aggregation, APIV provides a theoretically grounded and stable positional prior. We do not use learned positional weighting methods such as trainable position embeddings, as they tend to overfit spurious positional patterns in small, imbalanced biomedical datasets. In contrast, APIV avoids extra parameters, improves generalization, and enhances robustness in complex sentences with many drug entities.

#### 2.3.3. Molecular Graph Neural Networks

Traditional GNNs are computationally efficient but struggle to model complex molecular structures [26]. In many drug molecules, critical remote interactions occur between distant atoms or functional groups (e.g., hydrophobic interactions, hydrogen bonds, π–π stacking), which strongly influence drug activity, metabolic pathways, and target binding. However, conventional GNNs only aggregate local neighbors and cannot effectively capture such long-range dependencies, leading to insufficient representation of structural semantics.

Notably, long-range interactions in molecular graphs are inherently sparse: only a small number of distal atom pairs carry meaningful chemical signals [26]. This sparsity makes sparse attention theoretically more suitable than full attention for molecular representation learning [23]. The ProbSparse mechanism automatically identifies chemically critical atoms or functional groups by selecting queries with high KL divergence while pruning irrelevant nodes.

**Figure 3:**
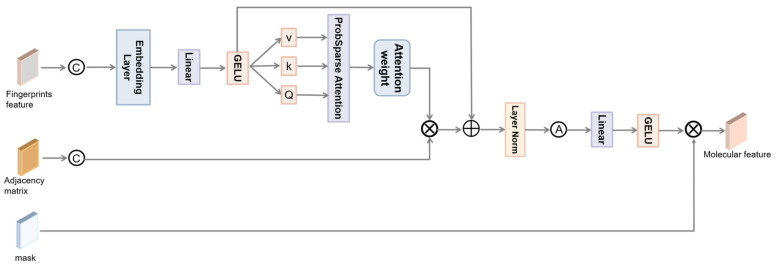
Molecular graph neural network incorporating ProbSparse self-attention mechanism.

To overcome these limitations, we introduce ProbSparse self-attention [23] and propose PS-AEGNN, a molecular graph neural network tailored for DDI extraction (Figure 3). By integrating local chemical structures and global semantic dependencies via sparse query–key interactions, PS-AEGNN accurately captures functional group activities, metabolic pathways, and distant structural relationships. This strengthens molecular representation and provides a reliable chemical basis for subsequent cross-modal alignment with textual semantics.

The drug molecular structures were obtained from the DrugBank database [27] as SMILES sequences and converted into graph structure representations (nodes represent atoms, and edges represent chemical bonds) using the RDKit toolkit(RDKit version 2022.09.5, RDKit open-source cheminformatics software, available at https://www.rdkit.org) [28]. The feature extraction method of Tsubaki et al. [29] was used to generate molecular fingerprint features that contain atom types, chemical bond types, and functional group identifiers. First, we represented each atom with a fingerprint vector, which was input into PS-AEGNN as an initial vector:

(11) $$m_{i}^{\left(0\right)}=embed\_fingerprint\cdot \left({fingerprints}_{i}\right)$$

where $m_{i}^{\left(0\right)}$ denotes the vector representation of the ith atom at atom layer 0, embed_fingerprint denotes the embedding layer, and ${fingerprints}_{i}$ denotes the fingerprint index corresponding to the i-th atom. In each layer, the node vector $m_{i}^{\left(l\right)}$ is updated as follows:

(12) $$m_{i}^{\left(l+1\right)}=f_{update}\left(m_{i}^{\left(l\right)}\left\{m_{i}^{\left(l\right)}|j\in N_{i}\right\}\right)$$

where $N_{i}$ is the set of neighboring atoms of the i-th atom. The classic GNNs have the flaw of “treating all neighbors equally” when aggregating neighbor information, making it impossible to discern the semantic contributions of critical chemical bonds. To address this, the ProbSparse self-attention method is presented, which uses a sparse query selection strategy to dynamically focus critical node connections. In the update function $f_{update}()$, we invoke the ProbSparse self-attention mechanism to select the most important neighbor information. Linear transformation and activation operations are performed on all node vectors in the l-th layer:

(13) $$h_{i}^{l}=ReLU\left(W_{fingerprint}^{l}\cdot m_{i}^{l}\right)$$

$W_{fingerprint}^{l}$ is the linearly transformed weight matrix of layer l, and ReLU is the activation function. For each query vector $q_{i}^{l}$, the KL dispersion concerning U is computed:

(14) $$KL\left(q_{i}||\;U\right)=\sum_{k=1}^{d}q_{i}^{k}log\frac{q_{i}^{k}}{U^{k}}\;$$

where *d* is the feature dimension, $q_{i}^{k}$ is the *k*-th dimensional component of the query vector, and $U^{k}$ is a value uniformly distributed in dimension k. The *u* key queries with the largest KL scatter are selected, and only their attentional weights are computed concerning all keys:

(15) $$\alpha_{ij}=\frac{exp\left(\frac{q_{i}^{l}\cdot k_{j}^{l}}{\sqrt{d_{k}}}\right)}{\sum_{j^{\prime }\epsilon S_{i}}exp\left(\frac{q_{i}^{l}\cdot k_{j^{\prime }}^{l}}{\sqrt{d_{k}}}\right)}$$

where $d_{k}$ is the dimension of the key vector, $q_{i}^{l}\;and{\;k}_{j}^{l}$ are the vectors obtained from the query and key-value pairs by linear transformation, respectively, and $S_{i}$ is a small subset of keys selected from all possible keys according to the sparse query selection algorithm. Neighbor value vectors are weighted and summed using attention weights, and the node representations are updated by combining the residual connections:

(16) $$a_{i}^{l}=\sum_{j\epsilon S_{i}}\alpha_{ij}v_{j}^{l},\;m_{i}^{\left(l+1\right)}=h_{i}^{l}+a_{i}^{l}$$

Ultimately, drug molecule vectors are generated by aggregating all atom vectors:

(17) $$h^{mol}=f\left(W_{output}\sum_{i=1}^{M}m_{i}^{L}+b_{output}\right)$$

where M is the number of atoms, L is the total number of layers of the network, and $W_{output}$ and $b_{output}$ are the linear transformation matrices and biases of the output layers, which transform the molecular graph into a semantic vector representation of fixed dimensions by summation aggregation and nonlinear transformation.

#### 2.3.4. Biomedical Entity Subgraph Feature Extractor

In the DDI extraction task, interactions between drug molecules are influenced not just by their chemical structural features, but also by intricate interactions between other biological entities. To fully mine and utilize such structural and semantic information, this study introduces a knowledge subgraph feature extractor, which aims to identify subgraph structures related to drug pairs in a large-scale biomedical knowledge graph and extract key semantic features therein, thereby providing structured knowledge inputs with semantic support for multimodal fusion. At the knowledge level, such biological entity subgraph characteristics provide the underlying mechanisms and contextual information of DDI occurrence, further improving the model’s ability to recognize the complicated interactions among medications.

The knowledge graph data in this study were obtained from several authoritative public biomedical databases, such as DrugBank [27], TWOSIDES, OFFSIDES [30], SIDER [31], SMPDB [32], DrugCentral [33], Entrez Gene [34], CTD [35], and so on. The nodes in the graph represent various types of biomedical entities, including drugs, targets, pathways, side effects, diseases, genes, and phenotypes. The edges indicate the interrelationships among these entities, such as the drug–target binding relationship and the drug–side effect association relationship.

**Figure 4:**
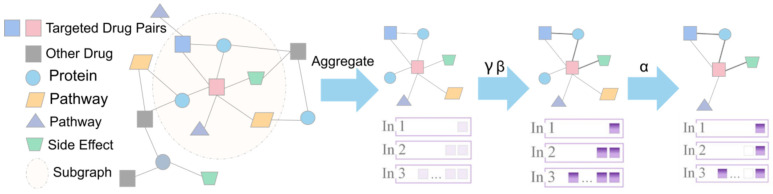
Process of SubAGCN for extracting drug pair-related subgraph information in knowledge graphs.

In this study, we introduced SubAGCN [17], a graph neural network model designed to extract information from drug-pair-related subgraphs within the knowledge graph. SubAGCN dynamically adjusts the weights of the edges in the subgraphs through a self-attention mechanism to retain information that is more useful for predicting DDI, as illustrated in Figure 4, considerably improving the performance of DDI relationship extraction.

Specifically, SubAGCN first calculates the weight of each edge using the self-attention module. For each edge (*i*,*j*) in the subgraph, its attention weight $\alpha_{i,j}$ is computed by the following equation:

(18) $$\alpha^{i,j}=Tanh(\frac{h_{\left(0\right)}^{j}W^{J}{\left(h_{\left(0\right)}^{i}W^{I}+r^{i,j}\right)}^{T}}{\sqrt{d^{k}}})\;$$

where $h_{\left(0\right)}^{j},h_{\left(0\right)}^{i}$ are the initial embeddings of node *i* and node *j*, respectively, $W^{I\;}$ and ${\;W}^{J}$ are the weight matrices of the linear layer, $r^{i,j}$ denotes the relationship between node i and node j, and $d^{k}$ is the dimension of the node’s input vector.

To filter out unimportant edges, SubAGCN introduces a threshold hyperparameter z. The weight $\tau_{i,j}$ of an edge (*i*,*j*) is set to 0 if $\alpha_{i,j}\leq z$, otherwise its weight is retained:

(19) $$\tau_{i,j}=\left\{\begin{array}{c} \alpha_{i,j},\;otherwise\\ 0,\;\alpha_{i,j}<=z \end{array}\right.\;$$

In this way, SubAGCN can retain key subgraph structures and remove edges that contribute less to DDI extraction and classification. When extracting subgraph features, SubAGCN employs a message-passing mechanism to aggregate node information. For each node *v*, the information of its neighbor node $b_{v}^{\left(t\right)}$ is calculated by the following equation:

(20) $$b_{\left(t\right)}^{v}=\sum_{u\in N_{v}}\tau_{\left(t\right)}^{u,v}(\gamma_{\left(t\right)}^{v}\left({h_{\left(t\right)}^{u}W}_{\left(t\right)}^{r}\right)+\beta_{\left(t\right)}^{v})\;$$

where $N_{v}$ is the set of all neighboring nodes of node *v* in the subgraph, and $W_{\left(t\right)}^{r}$ is the relationship weight matrix at the t-th level. Subsequently, SubAGCN updates the representation of node *v* by the following equation:

(21) $$h_{\left(t+1\right)}^{v}=ReLU(W_{\left(t\right)}^{self}h_{\left(t\right)}^{v}+b_{\left(t\right)}^{v})\;$$

where $W_{\left(t\right)}^{self}$ is the weight matrix used to transform the node embedding itself. Ultimately, SubAGCN generates a representation of the subgraph by using the following equation:

(22) $$h_{\left(t\right)}^{G_{Sub}}=Mean(W^{Sub}h_{\left(t\right)}^{i})$$

#### 2.3.5. Multi-Stage Adaptive Fusion Module

The multi-stage adaptive fusion module is responsible for integrating multimodal features from text features, molecular structures, and subgraphs of biomedical entities to achieve accurate classification of DDI. Our sequential design of “subgraph–molecule attention followed by text-guided gating” follows the information bottleneck principle [36] for multimodal fusion [37]: aligning auxiliary modalities first compresses task-irrelevant redundancy, while text-guided gating then preserves the most predictive cues. Underlying feature splicing is performed first. In the initial stage of multimodal fusion, text features $h^{text}$ extracted by BioBERT, molecular features $h^{mol}$ generated by molecular graph neural network, and subgraph features $h^{G_{Sub}}$ outputted by biomedical entity subgraph feature extractor are spliced. Specifically, the initial fusion feature $f_{0}$ is obtained:

(23) $$f_{0}=\left[h^{mol};h^{G_{Sub}};h^{text}\right]$$

where “;” denotes the vector splicing operation.

This was followed by mid-level cross-modal attention fusion. Subgraph–molecule cross-attention first establishes correlations between drug structures and biomedical entity knowledge, based on biomedical heterogeneous graph attention theory [17,38]. The weights of molecular structural features and subgraph features of biomedical entities are dynamically computed with text features as a guide. Taking molecular features as query Q and subgraph features as keys K and values *V*, the subgraph–molecule cross-modal attention is realized by dynamically focusing on key subgraph information with the help of attention weights in the following steps:

First, in the feature projection part, we project the molecular feature $h^{mol}$ and subgraph feature $h^{G_{Sub}}$ to dimension *d* through a linear layer:

(24) $$Q=Linear\left(h^{mol}\right)\epsilon R^{B\times d},K=Linear\left(h^{G_{Sub}}\right)\epsilon R^{B\times d},\;V=Linear\left(h^{G_{Sub}}\right)\epsilon R^{B\times d}$$

where B denotes the batch size. Attention scores are subsequently computed and normalized by dot product scaling:

(25) $$\alpha =Softmax(\frac{QK^{T}}{\sqrt{d_{k}}})$$

where $d_{k}$ is the dimension of the key vector, and the normalization factor $\sqrt{d_{k}}$ ensures that the gradient remains stable. Then, weighted aggregated value vectors are performed to obtain fusion features $h^{fused}$, which realize the interaction between molecular structure and biomedical knowledge:

(26) $$h^{fused}=attnV\epsilon R^{B\times d}$$

In order to realize text-guided fusion of textual semantics to subgraph–molecular features, a dynamic gating mechanism adjusts the fusion ratio of textual and graph features. Following the text-centric principle in biomedical information extraction [13], this mechanism dynamically allocates modality weights, with text as the dominant modality. The specific steps are as follows: The first projection is the subgraph–molecule fusion feature $h^{fused}$ to the text feature $d_{t}$ dimension:

(27) $$h^{proj}=Linear\left(h^{fused}\right)\epsilon R^{B\times d_{t}}$$

The gated signal generator (2-layer ReLU activation fully connected layer based on textual features) then processes the spliced text features $h^{text}$ and the projected subgraph–molecule features $h^{proj}$ to generate gated vectors of dimension [0,1]:

(28) $$G=Sigmoid\left(Linear[h^{text};h^{proj}]\right)\epsilon R^{B\times d_{t}}$$

Finally, a dynamically weighted fusion of text features and subgraph–molecule features is derived based on gating values:

(29) $$f_{1}=G⊙h^{proj}+\left(1-G\right)⊙T\epsilon R^{B\times d_{t}}$$

Grounded in gradient stability theory for deep networks [39], residual fusion with layer normalization preserves original features and mitigates modality conflicts; to avoid gradient vanishing and retain underlying details, this study implements high-level residual fusion and normalization, designing residual paths that superimpose the original spliced features onto the mid-level fused features:

(30) $$f_{residual}=f_{1}+f_{0}\left[:,:T_{fused}.shape\left[-1\right]\right]\epsilon R^{B\times d_{t}}\;$$

Finally, the training process is stabilized by layer normalization to output the final fused features:

(31) $$h_{final}=LayerNorm(f_{residual})\epsilon R^{B\times d_{t}}$$

The features include global guidance on textual semantics, local molecular structure attributes.

#### 2.3.6. Model Training

In the SemEval-2013 dataset [6], the Negative type accounts for more than 70% of the samples, while the Positive type accounts for less. As a result, the sample imbalance is avoided by employing multi-locus loss [40], a loss function that treats distinct DDI types as different weights using the following equation:

(32) $$MPL\left(p^{t}\right)=-\frac{\sum_{i=1}^{m}\alpha_{i}{\left(1-p^{t}\right)}^{\gamma }\log\left(p^{t}\right)}{m}\;$$

$m,\;p^{t},and\alpha_{i}$ represent the number of DDI kinds, the predicted probability of the DDI extraction model, and the weight of each DDI type in the following equation, respectively (*γ* = 2).

(33) $$\alpha_{i}=\frac{{Count}_{i}}{\sum_{i=1}^{m}{Count}_{i}}$$

${Count}_{i}\;$ stands for the number of the *i*-th type, and *m* for the number of distinct types.

#### 2.3.7. Evaluation Metrics

There are two common F1 scores: micro-f1 (${F1}_{micro}$) and macro-f1 (${F1}_{macro}$) scores. These two F1 score metrics have corresponding equations for precision and recall. The following equation is used to determine the micro-precision, recall, and F1 scores:

(34) $${Precision}_{micro}=\frac{\sum_{i=1}^{n}TP_{i}}{\sum_{i=1}^{n}TP_{i}+\sum_{i=1}^{n}FP_{i}}$$

(35) $${Recall}_{micro}=\frac{\sum_{i=1}^{n}TP_{i}}{\sum_{i=1}^{n}TP_{i}+\sum_{i=1}^{n}FN_{i}}$$

(36) $${F1}_{micro}=2\times \frac{{Recall}_{micro}{\times Precision}_{micro}}{{Recall}_{micro}{+Precision}_{micro}}$$

where $TP_{i},FP_{i},$ and $FN_{i}$ denote the number of instances of true positives, false positives, and false negatives for category i, respectively.

In this paper, we use the averages of macro precision and macro recall to calculate ${F1}_{macro}$, all of which are calculated as follows:

(37) $${Precision}_{macro}=\frac{\sum_{i=1}^{n}{Precision}_{i}}{n}$$

(38) $${Recall}_{macro}=\frac{\sum_{i=1}^{n}{Recall}_{i}}{n}\;$$

(39) $${F1}_{macro}=2\times \frac{{Recall}_{macro}{\times Precision}_{macro}}{{Recall}_{macro}{+Precision}_{macro}}$$

where:

(40) $${Precision}_{i}=\frac{TP_{i}}{TP_{i}+FP_{i}}$$

(41) $${Recall}_{i}=\frac{TP_{i}}{TP_{i}+FN_{i}}$$

N is the total number of categories, and i denotes the specified type.

## 3. Results

### 3.1. Comparison with Previous Models

To compare the performance of MultiMod-DDI with other state-of-the-art models, we evaluate our method using both macro-averaged and micro-averaged metrics. We benchmark the MultiMod-DDI model on the test dataset of the SemEval-2013 Task 9 dataset. 

As shown in Table 4 and Table 5, MultiMod-DDI significantly outperforms other models in four core metrics, namely ${F1}_{macro}$, ${F1}_{micro}$, ${Recall}_{micro}$, and ${Precision}_{macro}$, with 85.57 for ${F1}_{macro}$, 85.20 for ${F1}_{micro}$, 87.75 for ${Precision}_{macro}$, and 85.05 for ${Recall}_{micro}$. Comparing SRGU-CNN [9], TM-RNN [41] and other conventional machine learning models, models built using pre-training techniques typically outperform them, demonstrating the core benefits of pre-trained semantic representations in the comprehension of biological texts. Meanwhile, compared to SubGE-DDI [17], which also applies biomedical knowledge subgraphs, this method improves ${F1}_{micro}$ by 1.29% and ${F1}_{macro}$ by 3.57%. This improvement arises because MultiMod-DDI further incorporates molecular structure information to complement knowledge subgraph features, forming semantically complementary multimodal representations.

### 3.2. Performance of Multi-Classification and Ablation Analysis on Different DDI Types

The average F1 score on micro-averaged metrics for five-fold cross-validation is shown in Table 6. The experimental results reveal that the model that incorporates adaptive position vectors, molecular structure features, and knowledge subgraphs outperforms the baseline across all four kinds. This finding indicates that the proposed framework can effectively alleviate the category imbalance problem.

**Figure 5:**
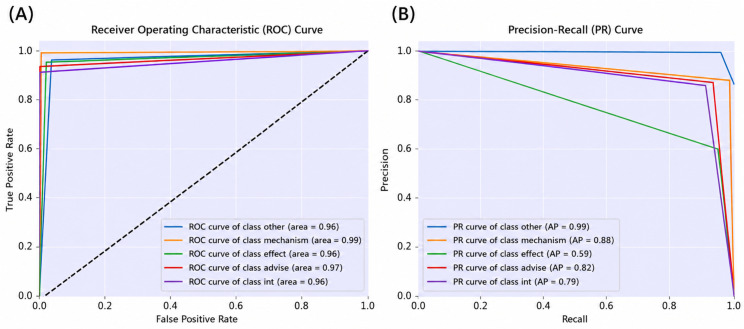
ROC curve and PR curve on SemEval-2013 Task 9 dataset, (**A**) ROC curve, (**B**) PR curve.

The Receiver Operating Characteristic (ROC) curve and Precision-Recall (PR) curve are shown in Figure 5. 

### 3.3. The Performance of Different Input Vectors

To quantify the contribution of each component to overall model performance, we use the BERT-only model as our baseline and conduct a series of ablation experiments. These experiments systematically incorporate adaptive position interaction vectors (APIV), molecular structure features (processed with either GNN or PS-AEGNN), and biomedical knowledge subgraphs (Subgraph). The results are summarized in Table 7.

When the model is based entirely on textual features (BERT-only), it achieves ${F1}_{macro}$ of 79.23% and ${F1}_{micro}$ of 78.31%, highlighting the limitations of unimodal approaches in capturing complicated DDI semantics. The integration of adaptive position interaction vectors significantly improves performance, increasing ${F1}_{macro}$ to 83.21% and ${F1}_{micro}$ to 82.63%. This demonstrates the importance of modeling long-distance semantic dependencies between drug entities in text. Similarly, incorporating knowledge subgraphs alone boosts ${F1}_{macro}$ to 83.95% and ${F1}_{micro}$ to 82.96%, indicating that structured biomedical knowledge provides valuable context for DDI recognition. In contrast, adding molecular structure features alone results in more modest improvements. To assess the efficacy of various molecular feature processing algorithms, we compare the performance of GNN and PS-AEGNN. As shown in Table 4, when molecular structure features are incorporated using GNN, the model achieves ${F1}_{macro}$ of 79.92% and ${F1}_{micro}$ of 79.53%. In contrast, using PS-AEGNN results in ${F1}_{macro}$ of 80.46% and ${F1}_{micro}$ of 81.81%. When all components are integrated, the model reaches peak performance.

These results demonstrate the effectiveness of each component. To validate the practicality of our architecture, we analyze the computational overhead of the full MultiMod-DDI model. Under the experimental setting (32 GB vGPU, batch size = 16), the complete training pipeline (20 epochs) requires approximately 5 h, with a peak GPU memory consumption of 16.2 GB. Notably, the introduction of the graph-based modules adds only 8.4 M learnable parameters to the BioBERT backbone. This represents a negligible increase in model size (approx. 7.5%) relative to the baseline, yet yields a significant improvement in extraction accuracy. Furthermore, the model achieves an inference speed of 15.3 ms per sentence. These results confirm that our model achieves a favorable trade-off: it maintains high computational efficiency and a compact parameter footprint while delivering superior performance.

### 3.4. Ablation on Intensity Ranges of Adaptive Position Interaction Vector

To validate the rationality of the manually set intensity range (0.9–1.1) in the adaptive position interaction vector, we conduct an ablation study over four commonly used candidate intervals while keeping other modules unchanged. The intensity ranges and corresponding performance are listed in Table 8.

As shown, the model achieves the highest *F1_macro_* (85.57%) and *F1_micro_* (85.20%) when the intensity range is set to 0.9–1.1. Lower intervals (0.5–0.7, 0.7–0.9) fail to sufficiently enhance the semantic contribution around drug entities, while an overly large range (1.1–1.3) introduces excessive weight fluctuation and degrades robustness. These results empirically confirm that the 0.9–1.1 setting is optimal and well-justified.

### 3.5. The Performance of Different Feature Fusion Strategies

To evaluate the effectiveness of different multimodal fusion strategies, we designed four fusion paradigms and compared their performance, as summarized in Table 9. All methods share the same baseline: initial concatenation of text, molecular structure, and knowledge subgraph features, followed by a high-level residual connection to preserve original information. The key distinction among them lies in the post-concatenation processing steps. The multi-stage adaptive fusion module sequentially performs subgraph–molecule cross-attention and text-guided gating. This layered “filter associations first, then align semantics” design effectively enhances multimodal complementarity while avoiding semantic conflicts caused by naive concatenation. Experimental results demonstrate that this approach achieves the best overall performance. Therefore, multi-stage adaptive fusion was selected.

**Figure 6:**
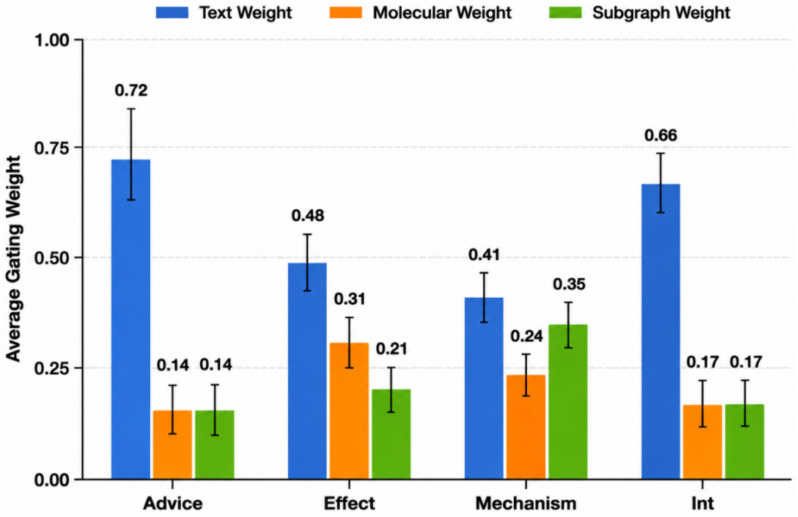
Distribution of average gating weights across modalities and DDI categories.

To further verify the adaptive modality selection ability of the text-guided gating mechanism, we computed the average gating weights for text, molecular structure, and knowledge subgraph modalities across different DDI types, as visualized in Figure 6. The results demonstrate that our model can automatically prioritize the most informative modality based on the characteristics of each interaction type.

### 3.6. Error Analysis

**Figure 7:**
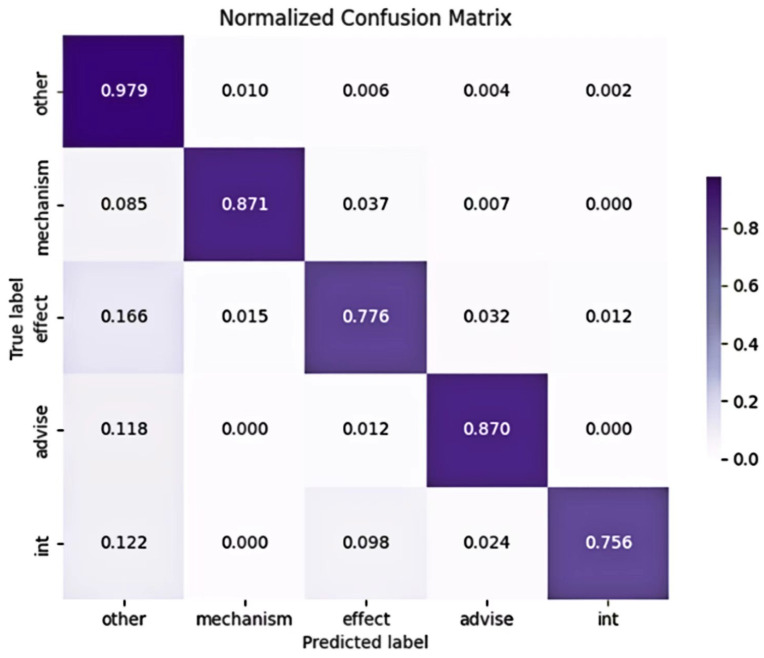
The confusion matrix of the results of our model.

Despite outperforming previous techniques, our model exhibits specific error patterns. As shown in the confusion matrix (Figure 7), the “Int” category suffers from low accuracy, often being misclassified as negative. This difficulty arises from both data scarcity and the nature of “Int” sentences, which only vaguely acknowledge an interaction without detailed mechanisms or effects. Consequently, the molecular and subgraph features—rich in mechanistic evidence—struggle to provide discriminative signals for this specific class, sometimes biasing predictions toward content-rich categories like “Mechanism”.

To ensure evaluation fairness, we applied resampling only to the training set. Consequently, the test set retains a long-tailed distribution, with negative samples comprising 83.0% of the data and positive samples only 17.0%. The “Int” category is particularly scarce, representing just 17.7% of positives. Oversampling this category risks memorization of superficial co-occurrence patterns due to limited linguistic diversity, potentially harming generalization.

Despite this challenge, our model outperforms state-of-the-art methods in the “Int” category. We attribute this to two key components: first, the knowledge subgraph supplements textual evidence with indirect biomedical associations, providing additional contextual cues even when sentence-level descriptions are vague; second, the adaptive position interaction vector captures implicit long-distance dependencies between drug entities, helping the model recognize weak interaction signals that would otherwise be missed. Together, these mechanisms enable robust performance despite the inherent difficulties of the “Int” category.

Beyond category-level imbalance, the introduction also identifies a structurally distinct difficulty: sentences containing multiple drug entities can mislead models that rely solely on surface co-occurrence. To evaluate how our model handles this challenge, we stratified the test set according to the number of drug mentions per sentence. Sentences were grouped into three strata: Simple (exactly 2 mentions, i.e., only the target drug pair), Medium (3 mentions), and Complex (≥4 mentions). Table 10 reports the *F1_macro_* scores of three model configurations on each stratum.

The BERT-only baseline suffers a severe performance drop (10.78 points) on Complex sentences, confirming that shallow models are easily distracted by irrelevant drug entities. In contrast, adding the adaptive position interaction vector (APIV) significantly boosts robustness, yielding a 9.14-point gain on Complex sentences—far exceeding the improvement on Simple sentences (+5.42 points). This demonstrates APIV’s specific effectiveness in suppressing “bystander drug” noise. The full MultiMod-DDI model further improves performance to 82.07%, showing that multimodal evidence complements positional enhancement. Residual errors often involve long sequences or anaphoric references, but these results empirically validate that APIV directly addresses the multi-entity challenge.

**Figure 8:**
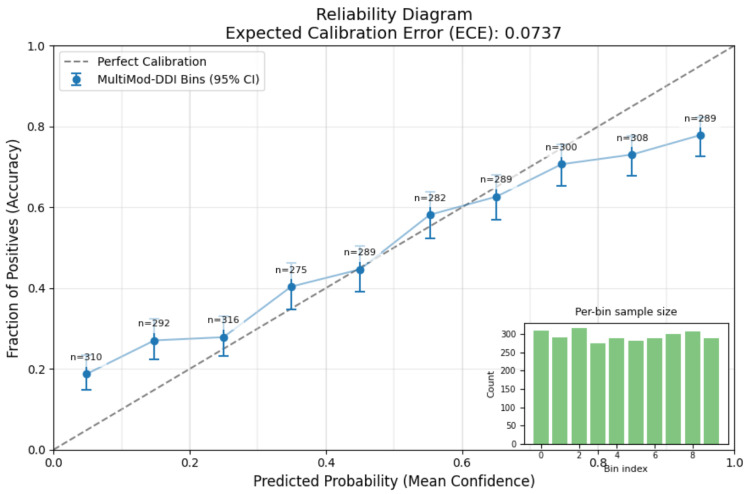
Reliability diagram for MultiMod-DDI calibration.

To further evaluate the reliability of MultiMod-DDI predictions, we perform a calibration analysis on the test set (Figure 8). The model achieves an ECE of 0.0737 (7.37%), indicating acceptable calibration. The reliability diagram shows that predicted confidence aligns well with empirical accuracy; narrow 95% confidence intervals and balanced per-bin sample sizes (275–316) ensure statistical stability. This analysis complements our error case studies, confirming that the model’s confidence estimates are generally reliable.

### 3.7. Case Study

**Figure 9:**
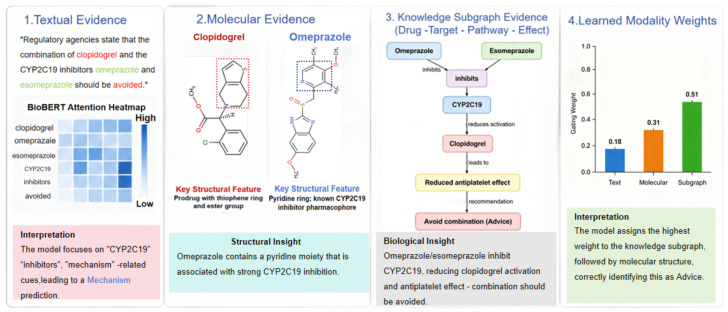
Multi-modal correction of a text-only DDI misclassification.

To illustrate how the combination of molecular graphs and knowledge subgraphs improves DDI extraction beyond text-only models, we present a representative test instance where the BERT-only model produces an incorrect classification, but our full MultiMod-DDI model yields the correct label. We detail how each modality contributes to the correction in Figure 9.

Despite overall improvement, multimodal evidence can occasionally mislead. First, molecular structural similarity may induce false mechanistic inference. When drug pairs share similar scaffolds but have different interaction mechanisms, the molecular encoder overweights structural resemblance, promoting incorrect “Mechanism” labels. Second, knowledge subgraphs can introduce distracting associations. For weakly informative sentences, the subgraph encoder sometimes prioritizes irrelevant target and pathway connections, pulling predictions toward “Effect” or “Mechanism” instead of the correct “Int” or “Advice”. These cases show that relying solely on molecular or subgraph evidence risks errors. Our multi-stage adaptive fusion module mitigates this by conditioning non-text modalities on textual context, though residual errors persist in highly ambiguous sentences. This highlights the value of context-guided multimodal alignment.

### 3.8. External Validation on DDIExtraction 2011

We further adopt DDIExtraction 2011 [48] as an independent external benchmark for external validation. This dataset is an early standard challenge corpus for drug–drug interaction extraction, constructed from DrugBank and biomedical literature, and has been widely used to evaluate the generalization ability of DDI extraction models.

We applied our pre-trained model directly to the DDIExtraction 2011 test set. As shown in Table 11, our model achieves a Macro-F1 score of 82.65 and a Micro-F1 score of 83.41. These results confirm that the multimodal evidence chain (molecular structure and biomedical subgraph) effectively enhances the model’s ability to generalize across different data sources and annotation standards.

## 4. Discussion

MultiMod-DDI’s contribution lies not in integrating existing modules, but in three non-trivial, task-specific adaptations. (1) PS-AEGNN adapts sparse ProbSparse attention to molecular graphs, capturing long-range chemical dependencies without quadratic complexity. (2) The adaptive position vector uses a dynamically calibrated intensity range (0.9–1.1), not a generic relative encoding, to suppress bystander-drug interference. (3) The fusion module enforces a sequential order (subgraph–molecule attention, then text-guided gating) motivated by the information bottleneck principle. These designs are purpose-built for multi-drug, multi-interaction sentences and are not interchangeable with off-the-shelf components. The result is a semantically coherent joint representation achieved through correlation learning rather than causal mechanistic reasoning.

The ablation results show that full integration of molecular, subgraph, and text features raises *F1_micro_* from 82.92% to 85.20%, with a slight *P_micro_* drop but a larger *R_micro_* gain. Raw molecular features can introduce structural redundancy, triggering false positives for similar-looking non-interacting pairs. Our fusion module tames this noise by conditioning molecular representations on biological subgraphs and textual context, thereby improving recall-oriented utility.

Despite its strong performance, the model has limitations. First, the inherent class imbalance in the dataset leads to suboptimal recall for the “Int” category. To address this, we employ a multi-focal loss with class-specific weights, not merely basic resampling. Nevertheless, further improvements could be achieved through contrastive learning or synthetic data generation. Second, the multimodal architecture increases computational complexity. Future work may explore model compression techniques such as pruning or quantization.

## 5. Conclusions

This study proposes MultiMod-DDI for drug–drug interaction extraction. Rather than simply integrating off-the-shelf components, the methodological novelty lies in three task-specific adaptations: (1) PS-AEGNN adapts ProbSparse attention to molecular graphs for sparse long-range dependency capture; (2) an adaptive position vector with calibrated intensity range (0.9–1.1) suppresses bystander-drug noise; and (3) a sequentially ordered fusion module (subgraph–molecule attention followed by text-guided gating) follows the information bottleneck principle. These components jointly capture cross-modal correlations that improve DDI extraction accuracy.

The adaptive position interaction vector enhances the modeling of long-range semantic dependencies among drug entities by dynamically optimizing positional attention weights. The introduced PS-AEGNN, embedded with the ProbSparse self-attention mechanism, accurately captures long-range interactive relationships among molecular functional groups and atomic structures. Furthermore, the multi-stage adaptive fusion module realizes deep cross-modal semantic alignment through text-guided gating and subgraph–molecule cross-attention, which further improves the overall performance of DDI classification.

Experimental results on the SemEval-2013 Task 9 dataset show that MultiMod-DDI achieves an *F1_macro_* of 85.57% and an *F1_micro_* of 85.20%, with outstanding performance on the “Mechanism” and “Advice” categories. This work demonstrates that structured multimodal fusion—integrating molecular structure features, biomedical knowledge subgraphs, and textual semantics through a staged alignment process—substantially improves DDI extraction accuracy compared to unimodal or naïvely concatenated approaches. The adaptive position interaction vector and PS-AEGNN provide richer representations of drug entities and their chemical properties, while the multi-stage fusion module learns cross-modal correlations that help resolve ambiguities in complex sentences. Future work could extend this framework toward true mechanistic reasoning by incorporating explicit pharmacokinetic parameters, causal pathway modeling, or symbolic knowledge about interaction mechanisms.

## Figures and Tables

**Table 1:** Statistics of the SemEval-2013 dataset.

	Train		Test	
	Drugbank	Medline	Drugbank	Medline
Documents	572	142	158	33
Sentences	5675	1301	973	326
Drug pairs	26,005	1787	5265	451
Negative pairs	22,216	1555	4381	356
Positive pairs	3789	232	884	95
Mechanism	1257	62	278	24
Effect	1535	152	298	62
Advice	818	8	214	7
Int	179	10	94	2

**Table 2:** Examples of preprocessing.

Mention1	Mention2	Preprocessed Input Sentence
Antacids	pseudoephedrine	DRUG1 increases the rate of absorption of DRUG2, while DRUGOTHER decreases it.
Antacids	kaolin	DRUG1 increases the rate of absorption of DRUGOTHER, while DRUG2 decreases it.
pseudoephedrine	kaolin	DRUGOTHER increases the rate of absorption of DRUG1, while DRUG2 decreases it.

**Table 3:** Data of interaction information between entities as a percentage of total data.

Relation	Train	Test	All
Mechanism	0.89	0.92	0.90
Effect	0.87	0.88	0.88
Advice	0.85	0.85	0.85
Int	0.84	0.86	0.85

**Table 4:** Comparison with other methods (macro-averaged measures).

Methods	Precision	Recall	F1-Score
TM-RNN (2019) [41]	74.11 ± 1.02	70.82 ± 1.15	72.43 ± 0.98
SciBert (2019) [42]	81.34 ± 0.98	75.06 ± 1.21	78.07 ± 1.05
BioBert (2019) [13]	85.87 ± 0.65	73.58 ± 1.42	79.23 ± 0.87
PubmedBert (2021) [18]	83.56 ± 0.72	77.97 ± 1.08	78.20 ± 0.92
DDIE-KGE-MFL (2022) [15]	86.14 ± 0.58	77.41 ± 1.33	81.54 ± 0.76
3DGT-DDI (2022) [43]	80.78 ± 0.89	**86.36 ± 0.64**	83.47 ± 0.68
IMSE (2022) [16]	85.03 ± 0.77	78.64 ± 1.26	80.82 ± 0.82
IK-DDI (2023) [44]	85.89 ± 0.69	73.46 ± 1.53	79.19 ± 0.91
HKG-DDIE (2023) [38]	84.99 ± 0.74	78.93 ± 1.19	80.59 ± 0.79
SubGE-DDI (2024) [17]	83.27 ± 0.88	80.19 ± 0.97	82.52 ± 0.73
ClinicalBERT (2025) [45]	81.54 ± 0.95	80.25 ± 0.92	81.23 ± 0.81
CNN-DDI (2025) [46]	80.50 ± 1.08	81.80 ± 0.85	82.68 ± 0.75
ours	**87.75 ± 0.54**	86.31 ± 0.48	**85.57 ± 0.42**

The bold font indicates the best of all results.

**Table 5:** Comparison with other methods (micro-averaged measures).

Methods	Precision	Recall	F1-Score
SciBert (2019) [42]	83.53 ± 0.92	75.80 ± 1.18	79.48 ± 1.02
BioBert (2019) [13]	80.57 ± 1.05	81.14 ± 0.83	80.85 ± 0.74
SRGU-CNN (2020) [9]	76.19 ± 1.21	73.34 ± 1.21	74.74 ± 1.15
PubmedBert (2021) [18]	85.04 ± 0.85	76.87 ± 1.22	80.75 ± 0.95
DDIE-DESC-MOL (2021) [47]	85.36 ± 0.72	82.83 ± 0.79	84.08 ± 0.63
DDIE-KGE-MFL (2022) [15]	85.77 ± 0.68	81.49 ± 1.01	83.58 ± 0.71
IMSE (2022) [16]	79.38 ± 0.71	82.21 ± 0.88	80.77 ± 0.86
HKG-DDIE (2023) [38]	86.25 ± 0.61	82.56 ± 0.94	84.36 ± 0.59
SubGE-DDI (2024) [17]	85.02 ± 0.79	82.82 ± 0.76	83.91 ± 0.66
ClinicalBERT (2025) [45]	82.12 ± 0.98	83.25 ± 0.69	82.65 ± 0.78
CNN-DDI (2025) [46]	84.87 ± 0.84	**86.81 ± 0.58**	83.81 ± 0.64
ours	**85.36 ± 0.51**	85.05 ± 0.55	**85.20 ± 0.46**

The bold font indicates the best of all results.

**Table 6:** Performance on individual DDI types in *F1_micro_*.

Method	DDI Types		
	Advice	Effect	Int.
Bert-only	85.50 ± 0.65	81.36 ± 0.30	65.52 ± 0.84
+Apiv	86.68 ± 1.20	82.68 ± 0.85	70.18 ± 0.67
+Subgraph	89.60 ± 0.74	83.52 ± 1.15	71.77 ± 1.03
+Mol	88.47 ± 0.56	81.56 ± 1.26	66.34 ± 0.86
+Apiv+Subgraph	89.76 ± 0.58	85.88 ± 0.27	73.47 ± 0.91
+Apiv+Subgraph+Mol	**90.48** ± **0.62**	**86.55** ± **0.45**	**77.16** ± **0.77**

The bold font indicates the best of all results. The “+” symbol in the table indicates that the corresponding feature is incorporated and added to the baseline model.

**Table 7:** Comparisons of both micro-averaged and macro-averaged metrics on different parts.

Model	P_macro_	R_macro_	F1_macro_	P_micro_	R_micro_	F1_micro_
Bert-only	85.87 ± 0.72	73.58± 1.34	79.23 ± 0.88	84.12 ± 0.65	73.26 ± 1.42	78.31 ± 0.91
+Mol (GNN)	86.10 ± 0.68	77.69 ± 1.21	79.92 ± 0.85	84.11 ± 0.62	78.46 ± 1.18	79.53 ± 0.83
+Mol (PS-AEGNN)	81.11 ± 0.94	82.60 ± 0.87	80.46 ± 0.78	84.93 ± 0.55	82.90 ± 0.79	81.81 ± 0.68
+APIV	88.15 ± 0.56	82.39 ± 0.91	83.21 ± 0.65	79.26 ± 1.08	86.29 ± 0.63	82.63 ± 0.72
+Subgraph	85.86 ± 0.69	79.96 ± 1.02	83.95 ± 0.71	83.68 ± 0.74	82.25 ± 0.88	82.96 ± 0.66
+Apiv+Subgraph	88.61 ± 0.52	82.85 ± 0.85	84.40 ± 0.62	**90.15 ± 0.42**	81.23 ± 0.96	82.92 ± 0.68
+Apiv+Subgraph+Mol (GNN)	86.84 ± 0.61	85.28 ± 0.63	85.45 ± 0.54	88.15 ± 0.48	82.39 ± 0.87	83.21 ± 0.59
+Apiv+Subgraph+Mol (PS-AEGNN)	**87.75 ± 0.56**	**86.31 ± 0.44**	**85.57 ± 0.48**	85.36 ± 0.53	**85.05 ± 0.58**	**85.20 ± 0.46**

The bold font indicates the best of all results. The “+” symbol in the table indicates that the corresponding feature is incorporated and added to the baseline model.

**Table 8:** Performance comparison under different APIV intensity ranges.

Intensity Range	P_macro_	R_macro_	F1_macro_	P_micro_	R_micro_	F1_micro_
0.5–0.7	84.36 ± 0.92	80.12 ± 1.08	82.14 ± 0.85	82.45 ± 0.96	81.11 ± 1.02	81.77 ± 0.88
0.7–0.9	85.71 ± 0.74	81.73 ± 0.91	83.68 ± 0.72	83.69 ± 0.78	82.74 ± 0.85	83.21 ± 0.68
0.9–1.1	**87.75 ± 0.48**	**86.31 ± 0.56**	**85.57 ± 0.44**	**85.36 ± 0.53**	**85.05 ± 0.58**	**85.20 ± 0.46**
1.1–1.3	86.42 ± 0.65	81.90 ± 0.82	84.12 ± 0.62	84.11 ± 0.69	83.42 ± 0.76	83.76 ± 0.60

The bold font indicates the best of all results.

**Table 9:** Comparisons of *F1_macro_* and other metrics on different parts.

Model	P_macro_	R_macro_	F1_macro_	P_micro_	R_micro_	F1_micro_
Direct splicing	86.17 ± 0.71	85.13 ± 0.78	84.63 ± 0.68	82.93 ± 0.92	85.26 ± 0.92	82.72 ± 0.75
Only subgraph–molecule attention	84.31 ± 0.85	87.06 ± 0.62	84.70 ± 0.71	80.39 ± 1.04	87.27 ± 0.58	83.21 ± 0.68
Only text-guided gating	82.87 ± 0.94	88.43 ± 0.55	84.81 ± 0.73	87.29 ± 0.66	82.73 ± 0.82	83.08 ± 0.70
Multi-stage adaptive fusion	**87.75 ± 0.48**	**86.31 ± 0.56**	**85.57 ± 0.44**	**85.36 ± 0.53**	**85.05 ± 0.58**	**85.20 ± 0.46**

The bold font indicates the best of all results.

**Table 10:** Average modality gating weights for different DDI types.

Drug Mentions	Proportion in Test Set	BERT-Only	BERT + APIV	Full MultiMod-DDI
Simple (exactly 2)	17.2%	81.23 ± 0.85	86.65 ± 0.62	**88.41 ± 0.46**
Medium (3)	25.8%	76.58 ± 0.92	84.97 ± 0.68	**86.23 ± 0.52**
Complex (≥4)	57.0%	70.45 ± 1.08	79.59 ± 0.75	**82.07 ± 0.58**

The bold font indicates the best of all results.

**Table 11:** Performance on the external dataset DDIExtraction 2011.

Model	Macro-F1	Micro-F1
MultiMod-DDI	82.65 ± 0.56	83.41 ± 0.75

## Data Availability

The source code and data are available at https://github.com/YLM-0828/MultiMod-DDI (accessed on 20 May 2026).

## Abbreviations

DDIs	Drug–Drug Interactions
CRF	Conditional Random Fields
SVM	Support Vector Machine
CNNs	Convolutional Neural Networks
RNNs	Recurrent Neural Networks
LSTM	Long Short-Term Memory
BiLSTM	Bidirectional Long Short-Term Memory
GRUs	Gated Recurrent Units
GNN	Graph Neural Network
PS-AEGNN	ProbSparse Attention-based Graph Neural Network
APIV	Adaptive Position Interaction Vector
SubAGCN	Subgraph Attention Graph Convolutional Network
MPL	Multi-Focal Loss
ROC	Receiver Operating Characteristic
PR	Precision Recall
KL	Kullback–Leibler Divergence

## References

[1] Trumic E., Pranjic N., Begic L., Becic F. (2012). Prevalence of Polypharmacy and Drug Interaction Among Hospitalized Patients: Opportunities and Responsabilities in Pharmaceutical Care. Mater. Socio Medica.

[2] Lin X., Dai L., Zhou Y., Yu Z.-G., Zhang W., Shi J.-Y., Cao D.-S., Zeng L., Chen H., Song B. (2023). Comprehensive evaluation of deep and graph learning on drug–drug interactions prediction. Brief. Bioinform..

[3] Khandeparkar A., Rataboli P.V. (2017). A study of harmful drug–drug interactions due to polypharmacy in hospitalized patients in Goa Medical College. Perspect. Clin. Res..

[4] Vilar S., Friedman C., Hripcsak G. (2018). Detection of drug–drug interactions through data mining studies using clinical sources, scientific literature and social media. Brief. Bioinform..

[5] Perera N., Dehmer M., Emmert-Streib F. (2020). Named Entity Recognition and Relation Detection for Biomedical Information Extraction. Front. Cell Dev. Biol..

[6] Segura-Bedmar I., Herrero-Zazo M. (2013). SemEval-2013 Task 9: Extraction of Drug-Drug Interactions from Biomedical Texts (DDIExtraction 2013). Proceedings of the Seventh International Workshop on Semantic Evaluation (SemEval 2013).

[7] Zhao S., Su C., Lu Z., Wang F. (2021). Recent advances in biomedical literature mining. Brief. Bioinform..

[8] Kolchinsky A., Lourenço A., Li L., Rocha L.M. (2013). Evaluation of linear classifiers on articles containing pharmacokinetic evidence of drug-drug interactions. Biocomputing.

[9] Wu H., Xing Y., Ge W., Liu X., Zou J., Zhou C., Liao J. (2020). Drug-drug interaction extraction via hybrid neural networks on biomedical literature. J. Biomed. Inform..

[10] Hernandez-Lemus E., Bhasuran B., Natarajan J. (2018). Automatic extraction of gene-disease associations from literature using joint ensemble learning. PLoS ONE.

[11] Liu S., Tang B., Chen Q., Wang X. (2016). Drug-Drug Interaction Extraction via Convolutional Neural Networks. Comput. Math. Methods Med..

[12] Zheng W., Lin H., Luo L., Zhao Z., Li Z., Zhang Y., Yang Z., Wang J. (2017). An attention-based effective neural model for drug-drug interactions extraction. BMC Bioinform..

[13] Lee J., Yoon W., Kim S., Kim D., Kim S., So C.H., Kang J. (2020). BioBERT: A pre-trained biomedical language representation model for biomedical text mining. Bioinformatics.

[14] Devlin J., Chang M.-W., Lee K., Toutanova K. (2019). BERT: Pre-training of Deep Bidirectional Transformers for Language Understanding. Proceedings of the 2019 Conference of the North American Chapter of the Association for Computational Linguistics: Human Language Technologies.

[15] Asada M., Miwa M., Sasaki Y., Arne E. (2021). Using drug descriptions and molecular structures for drug–drug interaction extraction from literature. Bioinformatics.

[16] Duan B., Peng J., Zhang Y. (2022). IMSE: Interaction information attention and molecular structure based drug drug interaction extraction. BMC Bioinform..

[17] Shi Y., He M., Chen J., Han F., Cai Y. (2024). SubGE-DDI: A new prediction model for drug-drug interaction established through biomedical texts and drug-pairs knowledge subgraph enhancement. PLoS Comput. Biol..

[18] Gu Y., Tinn R., Cheng H., Lucas M., Usuyama N., Liu X., Naumann T., Gao J., Poon H. (2021). Domain-Specific Language Model Pretraining for Biomedical Natural Language Processing. ACM Trans. Comput. Healthc..

[19] Tanvir F., Saifuddin K.M., Akbas E. (2022). DDI prediction via heterogeneous graph attention networks. arXiv.

[20] Hu B., Yu Z., Li M. (2024). MPHGCL-DDI: Meta-Path-Based Heterogeneous Graph Contrastive Learning for Drug-Drug Interaction Prediction. Molecules.

[21] Tanvir F., Islam M.I.K., Akbas E. Predicting drug-drug interactions using meta-path based similarities. Proceedings of the 2021 IEEE Conference on Computational Intelligence in Bioinformatics and Computational Biology (CIBCB).

[22] Yang L., Shi Y., Han F., Cai Y. MultiMod-DDI: A drug-drug interaction prediction model based on the ternary evidence chain of “molecular structure–biological entities–DDI text”. Proceedings of the 2nd International Conference on Biomedical Engineering and Medical Devices (ICBEMD 2026).

[23] Zhou H., Zhang S., Peng J., Zhang S., Li J., Xiong H., Zhang W. Informer: Beyond Efficient Transformer for Long Sequence Time-Series Forecasting. Proceedings of the AAAI Conference on Artificial Intelligence.

[24] Shaw P., Uszkoreit J., Vaswani A. (2018). Self-attention with relative position representations. arXiv.

[25] Provoost T., Moens M.F. (2015). Semi-supervised learning for the BioNLP gene regulation network. BMC Bioinform..

[26] Fang Z., Yan Q. (2025). Leveraging Persistent Homology Features for Accurate Defect Formation Energy Predictions via Graph Neural Networks. Chem. Mater..

[27] Wishart D.S., Feunang Y.D., Guo A.C., Lo E.J., Marcu A., Grant J.R., Sajed T., Johnson D., Li C., Sayeeda Z. (2018). DrugBank 5.0: A major update to the DrugBank database for 2018. Nucleic Acids Res..

[28] Landrum G. (2016). RDKit: Open-Source Cheminformatics Software. http://www.rdkit.org.

[29] Tsubaki M., Tomii K., Sese J., Wren J. (2019). Compound–protein interaction prediction with end-to-end learning of neural networks for graphs and sequences. Bioinformatics.

[30] Tatonetti N.P., Ye P.P., Daneshjou R., Altman R.B. (2012). Data-Driven Prediction of Drug Effects and Interactions. Sci. Transl. Med..

[31] Kuhn M., Letunic I., Jensen L.J., Bork P. (2016). The SIDER database of drugs and side effects. Nucleic Acids Res..

[32] Jewison T., Su Y., Disfany F.M., Liang Y., Knox C., Maciejewski A., Poelzer J., Huynh J., Zhou Y., Arndt D. (2014). SMPDB 2.0: Big Improvements to the Small Molecule Pathway Database. Nucleic Acids Res..

[33] Avram S., Bologa C.G., Holmes J., Bocci G., Wilson T.B., Nguyen D.-T., Curpan R., Halip L., Bora A., Yang J.J. (2021). DrugCentral 2021 supports drug discovery and repositioning. Nucleic Acids Res..

[34] Brown G.R., Hem V., Katz K.S., Ovetsky M., Wallin C., Ermolaeva O., Tolstoy I., Tatusova T., Pruitt K.D., Maglott D.R. (2015). Gene: A gene-centered information resource at NCBI. Nucleic Acids Res..

[35] Davis A.P., Grondin C.J., Johnson R.J., Sciaky D., Wiegers J., Wiegers T.C., Mattingly C.J. (2021). Comparative Toxicogenomics Database (CTD): Update 2021. Nucleic Acids Res..

[36] Tishby N., Zaslavsky N. (2015). Deep learning and the information bottleneck principle. arXiv.

[37] Baltrušaitis T., Ahuja C., Morency L.-P. (2019). Multimodal machine learning: A survey and taxonomy. IEEE Trans. Pattern Anal. Mach. Intell..

[38] Asada M., Miwa M., Sasaki Y., Lu Z. (2023). Integrating heterogeneous knowledge graphs into drug–drug interaction extraction from the literature. Bioinformatics.

[39] He K., Zhang X., Ren S., Sun J. Identity mappings in deep residual networks. Proceedings of the European Conference on Computer Vision (ECCV).

[40] Lin T.-Y., Goyal P., Girshick R., He K., Dollar P. (2020). Focal Loss for Dense Object Detection. IEEE Trans. Pattern Anal. Mach. Intell..

[41] Liu J., Huang Z., Ren F., Hua L. (2019). Drug-Drug Interaction Extraction Based on Transfer Weight Matrix and Memory Network. IEEE Access.

[42] Beltagy I., Lo K., Cohan A. SciBERT: A Pretrained Language Model for Scientific Text. Proceedings of the 2019 Conference on Empirical Methods in Natural Language Processing and the 9th International Joint Conference on Natural Language Processing (EMNLP-IJCNLP).

[43] He H., Chen G., Yu-Chian Chen C. (2022). 3DGT-DDI: 3D graph and text based neural network for drug–drug interaction prediction. Brief. Bioinform..

[44] Dou M., Ding J., Chen G., Duan J., Guo F., Tang J. (2023). IK-DDI: A novel framework based on instance position embedding and key external text for DDI extraction. Brief. Bioinform..

[45] Liu X., Liu H., Yang G., Jiang Z., Cui S., Zhang Z., Wang H., Tao L., Sun Y., Song Z. (2025). A generalist medical language model for disease diagnosis assistance. Nat. Med..

[46] Tahir M.T., Ibrahim M., Sarwar N., Irshad A., Atteia G. (2025). Enhanced drug-drug interaction extraction from biomedical text using deep learning-based sentence representations. Sci. Rep..

[47] Jin X., Sun X., Chen J., Sutcliffe R. Extracting Drug-drug Interactions from Biomedical Texts using Knowledge Graph Embeddings and Multi-focal Loss. Proceedings of the CIKM ‘22: Proceedings of the 31st ACM International Conference on Information & Knowledge Management.

[48] Segura-Bedmar I., Martínez P., Sánchez-Cisneros D. The 1st DDIExtraction-2011 challenge task: Extraction of Drug-Drug Interactions from biomedical texts. Proceedings of the 1st Challenge Task on Drug-Drug Interaction Extraction.

